# AI-driven solutions to improve safety and health: Application of the REDECA framework for agricultural tractor drivers

**DOI:** 10.1371/journal.pgph.0003543

**Published:** 2025-06-04

**Authors:** Negin Ashrafi, Sahar Yousefi, Guy Roger Aby, Salah F. Issa, Houshang Darabi, Kamiar Alaei, Greg Placencia, Maryam Pishgar

**Affiliations:** 1 Department of Industrial and Systems Engineering, University of Southern California, Los Angeles, California, United States of America; 2 Department of Agricultural and Biological Engineering, University of Illinois at Urbana-Champaign, Urbana, Illinois, United States of America; 3 Marshfield Clinic Health System, Marshfield, Wisconsin, United States of America; 4 Department of Mechanical and Industrial Engineering, University of Illinois at Chicago, Chicago, Illinois, United States of America; 5 Department of Health Science, California State University, Long Beach, Long Beach, California, United States of America; 6 Department of Industrial and Manufacturing Engineering, California State Polytechnic University Pomona, Pomona, California, United States of America; PLOS: Public Library of Science, UNITED STATES OF AMERICA

## Abstract

**Introduction:**

Despite tremendous efforts, including research, teaching, and extension, toward improving the safety of agricultural tractor drivers, the number of incidents related to agricultural tractor drivers has not declined. This evidence points out an urgent need to explore artificial intelligence (AI) solutions to improve the safety of tractor drivers.

**Methods:**

This paper uses 171 Fatality Assessment and Control Evaluation (FACE) reports related to tractor drivers and a new framework called Risk Evolution, Detection, Evaluation, and Control of Accidents (REDECA) to identify existing AI solutions, such as machine learning models for predictive maintenance, sensor-based monitoring, computer vision, and automated safety interventions, and specific areas where AI solutions are missed and can be developed to reduce incidents and recovery time. Fatality reports of tractor drivers were categorized into six main categories, including run over, pinned by/ Crushed and entanglement, fall, fire, roll over, and overturn. Each category was then subcategorized based on similarities of incident causes in the reports.

**Results:**

The application of the REDECA framework, which categorizes risk states into R1 (safe), R2 (hazard exposure), and R3 (incident), revealed potential AI solutions that could improve the safety of tractor drivers. In all categories, the REDECA framework lacks AI solutions for three elements, including the probability of reducing recovery time in R3, detecting changes between R2 and R3, and intervention to send workers to R2. Most of the categories were missing AI solutions for interventions to prevent entry to the R3 element of the REDECA. In addition, the fall, roll over, and overturn categories lacked AI intervention that minimized damage and recovery in R3.

**Conclusions:**

The outcome of this study shows an urgent need to develop AI solutions to improve tractor driver safety.

## 1. Introduction

Agriculture has the highest rates of fatality incidents in the US [1]. While the number of fatal incidents dropped from around 1000 cases in the early 1990s to less than 600 cases in 2019, this decline is attributed to both the reduction in the number of workers over the past 30 years and the introduction of more efficient machinery and systems that have improved safety [2]., this has been because the number of workers has dropped over 30 years. Because of more efficient machinery and systems [2]. Moreover, within all types of agricultural fatal injuries (tractor, roadway, grain bins, farm equipment, Terrain Vehicle (ATV), electrocution, animals, manure storage, and others), tractor-related injuries remain a leading cause of fatalities, accounting for approximately 90 deaths annually, primarily due to rollovers. Over the 20-year period from 1999 to 2019, 213 tractor-related fatalities were reported. While this may represent a smaller proportion of total agricultural fatalities (600–1,000 annually), these deaths are significant because they are highly preventable, underscoring the need for focused interventions to improve tractor safety [3]. the number of tractor-related injuries remains high, with 213 cases from 1999 to 2019 [3].

Over the past few years, several efforts have been made to improve the safety of agricultural workers. These include research, teaching, and extension. In the case of tractor-related injuries, for instance, the focus has been on improving the design and functionality of tractors, while the teaching and extension focus has been on providing farmers safe practices. Despite such efforts, fatal tractor-related incidents are common, indicating a need to explore different approaches to tackle tractor-related injuries.

AI has been applied to many domains. In 2019 alone, over 20,000 papers were published to show the application of AI in various industries [4]. Both academia and industries have used AI to address a variety of issues, including decision-making [5], environmental monitoring [6,7], operational cost reduction [8], and productivity [9]. Machine learning algorithms have also been used to detect vocal disorders in workers who frequently use their voices [10], and to detect indoor crews in the event of fire. Gomez-Gil et al. used EMG readings to steer a tractor with almost the same accuracy as manual steering [10]. Szczepaniak et al. developed models to assess the stability and steerability of agricultural machines that could be adapted to drivers’ characteristics to improve safety [11]. Sensors can measure vibrations experienced by farmers using agricultural aircraft. Tri-axial accelerometers were used to measure acceleration at the seat level [12]. Kociolek et al. showed that operators on quad bikes were exposed to head and neck vibration at higher than permissible levels of exposure [13]. Similarly, Calvo et al. used three different accelerometers to measure hand-to-arm vibration and repetitive action (OCRA) levels for farmers who used power tillers. The result indicated vibrational exposure far above acceptable exposure levels [14]. These studies highlight mounting evidence that AI technology can successfully detect, identify, and forecast unsafe behavior in potentially dangerous working environments.

The REDECA (Risk Evolution, Detection, Evaluation, and Control of Accidents) [15] framework theorizes how AI methods can anticipate and control the risk of exposure in a worker’s immediate environment. It is based on the Swiss cheese model [16], which illustrates how safety incidents occur due to failures in multiple layers of defense. REDECA extends this concept by incorporating AI-driven risk prediction, detection, and mitigation strategies. The framework defines three risk states: R1, where workers have minimal to no risk of exposure; R2, where exposure to hazards increases the risk of injury; and R3, where a harmful work-related event occurs. AI technologies play a crucial role in monitoring transitions between these states by predicting or detecting shifts in risk levels. Additionally, intervention strategies are implemented to either prevent adverse transitions or minimize the impact of an incident, ultimately enhancing workplace safety. For example, a tractor operating on flat, stable ground may rely on AI to monitor its speed, tilt, and proximity to obstacles, providing early warnings if deviations from safe conditions are detected. R2 indicates a state of increased hazard exposure, where risks are present, but no incident has yet occurred. In this state, AI systems play a critical role in detecting changes in conditions and alerting workers to take corrective actions. For instance, if a tractor begins operating on a slope, sensors may detect a higher tilt angle and increased instability, triggering real-time alerts to the driver. These alerts may also suggest specific interventions, such as reducing speed or adjusting the route. This proactive detection helps prevent incidents by returning the system to R1 or stabilizing it within R2. R3 occurs when an incident has taken place, such as a tractor rollover. In this state, the focus shifts to minimizing harm and accelerating recovery. AI systems activate protective mechanisms, such as deploying a rollover protection structure (ROPS) or locking brakes to stabilize the vehicle. Simultaneously, the system can notify emergency responders and provide critical information about the incident, expediting assistance. After the incident, the AI system analyzes what occurred, offering insights to prevent similar events in the future [15].

The REDECA framework integrates AI solutions to manage risks and transitions between states effectively. In this study, machine learning models analyze large datasets, such as incident reports, to identify patterns and predict the likelihood of transitions between R1, R2, and R3 [17]. Predictive analytics tools forecast hazardous conditions, allowing early interventions to prevent incidents [18]. Real-time sensor systems monitor operational and environmental parameters, such as tractor speed, tilt, and terrain, feeding data into AI models for continuous risk assessment [19]. Automated safety mechanisms, such as rollover protection structures (ROPS), leverage AI insights to minimize harm and accelerate recovery during incidents [20]. These AI technologies collectively enhance safety by addressing risks dynamically across all REDECA states.

**Fig 1:**
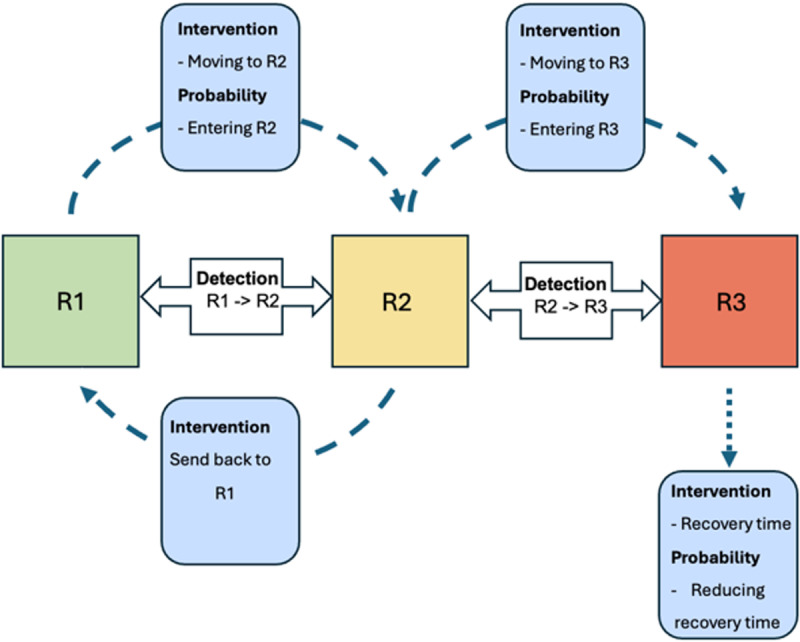
REDECA Framework: Risk States and Transitions: R1 is the state in which a worker has minimal to no risk of exposure. R2 indicates exposure to hazards and an increased risk of injury. R3 indicates a harmful work-related event happened. AI solutions can be applied at different stages: Blue boxes show technologies that can predict the probability of transitioning into the next states and show the intervention strategies to keep the worker safe or reduce the impact of a work-related event. White boxes are technologies that can detect transitions between the states.

For example, consider a tractor operator transitioning between these states. While operating on stable ground (R1), sensors monitor safe conditions. As the tractor approaches an incline (R2), the system detects increased risk and alerts the operator to take action. If these warnings are ignored and a rollover occurs (R3), protective mechanisms engage to minimize harm, and emergency services are notified. The AI system then evaluates the event, identifying ways to enhance safety measures. This example illustrates how REDECA integrates AI technologies—such as sensors, machine learning algorithms, and automated controls—to manage risks effectively at each stage. Fig 1 visually represents the framework, showing the risk states, transitions, and the role of AI-driven interventions.

The objectives of this paper are: (1) identify root causes of agricultural tractor driver incidents; (2) apply the REDECA framework to determine all steps/ stages involved before and after the occurrence of incidents; (3) determine existing AI solutions to reduce agricultural tractor driver incidents; (4) identifying opportunities for both industry and academia to propose new AI interventions to improve the safety of tractor drivers based on missing REDECA elements; and (5) provide good general practices to improve the safety of tractor drivers.

## 2. Materials and methods

Agriculture remains the most dangerous occupation in the U.S. [1]. Among all agricultural injuries, tractor-related injuries are the highest [3]. Several databases track all occupational incidents, such as Ag Injury News Clippings, the Fatality Assessment and Control Evaluation (FACE), and the Bureau of Labor (BOL) statistics. We studied FACE reports on fatal incidences among agricultural tractor drivers because they provide detailed case narratives, including contributing factors and recommendations for prevention, making them a valuable resource for understanding tractor-related fatalities [21] and were the most complete and comprehensive. This study uses de-identified, publicly available FACE reports from the CDC website. The data contain no personal identifiers and focus on aggregated case narratives, ensuring compliance with ethical standards. As no private information or direct interaction with individuals is involved, this study does not constitute human subject research under federal regulations (45 CFR 46) [22,23].

We used the following procedures to extract and analyze FACE report data. (1) We accessed FACE reports at national and state levels from the Center for Disease Control and Prevention (CDC) website, (2) using the keywords “agriculture” to find agricultural-related reports, (3) and “machine farming” to find machine-related cases. (4) We extracted and saved the reports to an Excel file. (5) We then identified all tractor-related cases by searching the Excel file for the keyword “tractor”. (6) In cases where national and state reports were identical, we analyzed the cases as a single incident. All reports were reviewed, and unwitnessed reports were excluded from analysis.

Further analysis (7) categorized reports based on types of tractor-related incidents: roll over, run over, overturn, pinned by, fall, and others that include fire, crashed incidents, as well as other types of incidents. Additionally, (8) in each category, reports with the same causes were sub-categorized together. Categories and subcategories are detailed in Table 1.

**Table 1:** Incident categories and sub-categories.

Category	Subcategories
**Run Over**	**Mechanical Failure:** Non-functional brake, Non-functional gear. **Driver Error:** Unsecured seat placement, Tractor left in gear with driver outside, Driver standing on ground while starting tractor, Driver falling off at high speed or while making a sharp turn, Driver fell off and tried to jump back on, Unintended door opening, Driver not using a seat belt (seizure-related), Poor driver visibility. **Environmental/Hazard Factors:** Tree stump obstruction, Hill-related roll-off, Water tank trailer weight & speed, Tractor engine slipping into gear at high RPM.
**Pinned By/Crushed** **&** **Entanglement**	**Collision with Obstacles:** Pinned between tractor and barn wall, Pinned against a tree, Pinned by truck bed attached to a tree. Semi-truck hitting tractor, Detached wagon crushing the user **Equipment Malfunction or Detachment:** Pinned while detaching attachment, Pinned when tractor drove into a ditch, Pinned by rolling hayrack. **Entanglement/Entrapment:** Shirt caught in auger (no guard), Hay baler left on slope causing entanglement
**Fall**	**Mechanical Failure:** Unattached seat, Tractor jack-knifing. **Driver Error or Health Issues:** Slipping on tractor step, High-speed incline fall (no seatbelt), Falling due to heart issues (no seatbelt), Losing control while driving. **Falling Objects:** Hay bale fell from elevated bucket.
**Fires**	**Fires:** Tractor hitting gas line (poor visibility)(move to collision with obstacles- pinned by), Vinyl shrouds catching fire, Tractor struck by a tree igniting.
**Roll Over & Overturn**	**Terrain & Slope Issues:** Sharp turn into a ditch, Steep roadside incline, Slope terrain & ditch, Hill-related overturns, Sliding down an embankment (wet/muddy/snowy conditions). **Load/Weight-Related:** Overweight hay bale on slope, Overweight objects attached to tractor, Raised bucket making a left turn on incline. Tree haul exceeding recommended height. **Mechanical or Operator Issues:** Foot slipping off clutch while pulling another tractor, Brakes not engaged (transmission in gear), Non-functional gear shift, Speeding tractor, Tractor wheel lifting off ground.

**Fig 2:**
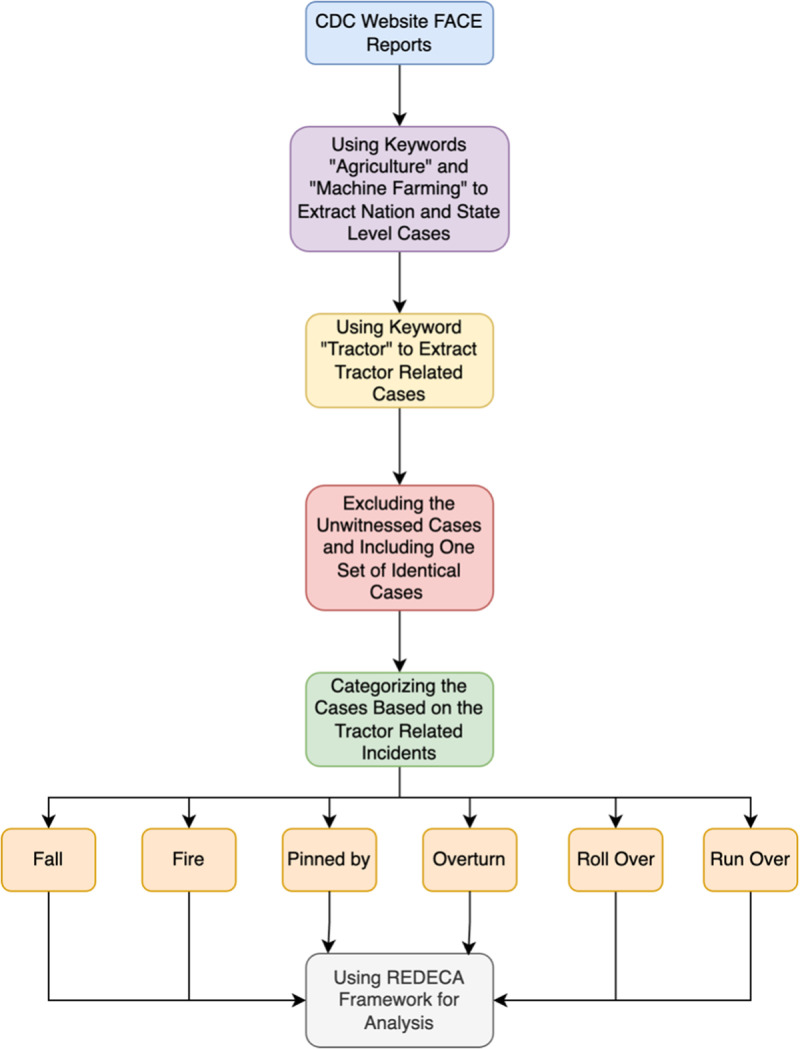
Overall view of the methodology.

We identified each state of work using the REDECA framework – from when tractor drivers started working until the incident happened (R1, R2, R3). We then indicated AI technologies that could predict the probability of transitioning among states, detect transitions among states, and indicate intervention strategies to keep the tractor drivers safe or reduce recovery times when incidents occur. Finally, we found existing AI technologies used by other industries and advised them to be used in the agriculture industry to improve the safety and health of tractor drivers. Fig 2 outlines our methodology.

The final step in this process, labeled ‘Using REDECA Framework for Analysis,’ involves categorizing each tractor-related incident into one of the three risk states: R1 (safe), R2 (hazard exposure), or R3 (incident). This categorization helps identify transitions between states and allows for the evaluation of AI-driven interventions aimed at preventing such transitions or minimizing the impact of incidents. For example, tractor operations on stable terrain (R1) transitioning to unstable conditions (R2) can be predicted using AI technologies, while incidents like rollovers (R3) can be mitigated with AI-driven safety measures. Furthermore, this analysis included assessing gaps in existing safety measures and identifying opportunities for AI-driven interventions. Each incident was evaluated for its associated risks, the conditions that led to transitions between states, and the presence or absence of preventive measures. By organizing the data in this manner, the REDECA framework provided a structured approach to understanding and mitigating risks in tractor-related incidents.

## 3. Results

From 442 initial agricultural cases of fatalities from FACE reports at national and state levels, we identified 188 tractor-related incidents using the keyword “tractor.” 9 cases were unwitnessed, and 16 were duplicates we combined into single cases. This left 171 cases for analysis.

We subcategorized 160 cases by the kind of tractor-related occurrence with similar causes (Table 1). We analyzed all cases using the REDECA framework (Fig 1) and contrasted each hazard against existing AI solutions in the areas of sensors, computer vision and predictive models (Table 2). The integration of sensors with AI technologies will play a crucial role in predictive safety applications. Wearable sensors, fatigue detection systems, and vehicle-based sensors generate real-time data that can be processed using AI algorithms to detect patterns indicative of potential hazards. These sensors, when combined with machine learning techniques, enhance early warning systems by predicting unsafe conditions and triggering timely interventions [24]. The synergy between AI models and sensor data enables a proactive approach to mitigating risks, ultimately contributing to improved safety and health outcomes for agricultural tractor drivers. **Table 2** presents a summary of various AI techniques used to mitigate tractor incidents, detailing how each technique can be applied within the REDECA framework to address different types of tractor-related incidents. In the realm of computer vision, AI-driven solutions such as drones, obstacle detection cameras, and risk map generation through deep learning models are integral to improving safety outcomes. Drones equipped with image-processing capabilities can monitor work environments in real-time, detecting hazardous conditions and providing valuable insights for preventive action. Obstacle detection cameras utilize convolutional neural networks (CNNs) to enhance situational awareness, identifying potential collisions and guiding drivers accordingly [25]. Additionally, risk maps generated via deep learning techniques process spatial and environmental data, enabling proactive safety interventions by highlighting high-risk areas [26]. The predictive models presented in the table focus on classification and feature selection techniques to enhance injury prediction for agricultural tractor drivers [27]. Injury classification models leverage machine learning algorithms to distinguish between injury and non-injury cases, employing supervised learning approaches. Feature importance analysis further refines these models by identifying key risk factors that contribute to unsafe conditions [27]. The evaluation of these models relies on metrics such as accuracy, precision, recall, and F1-score to ensure robust and reliable predictions.

**Table 2:** Summary of AI technologies in REDECA framework for different possible tractor incidents.

	Probability			Detection		Intervention				Resources
AI technologies	Entering R2	Entering R3	Recovery time	R1- > R2	R2- > R3	Stop- > R2	Stop- > R3	Move- > R1	Recovery time	
Wearable Sensors										
Smart watches	R	R							R,F,C,I,O	[28,29]
Sensors on Vehicle										
Workers fatigue detection sensors	O,C,F	O,C,F		O,C,F	O,C,F					[30,31]
Fall									F,C,R,O	[29]
Seatbelt sensors	R,C,R,O	R,C,R,O				R,C,R,O	R,C,R,O			[32]
High speed sensors	C.O,R	C,O,R				C,O,R	C,O,R			[33]
Computer vision										
Virtual reality for training drivers	R,F,I,C,O	R,F,I,C,O								[34,35]
Object detection cameras				P,R,C	P,R,C	P,R,C			P,R,C,O,I,F	[29,36]
Risk map prediction by using deep learning methods	C,O	C,O	C,O							[26]
Drones				I	I,C					[37]
Predicting injuries										
Injury/no injury classification	C,I,O	C,I,O								[27]
Feature importance	C,I,O	C,I,O								[27]

Abbreviations: R: Run over, O: Overturn and roll over, F: Fall, P: Pinned by, I: Fire, C: Crash.

The table reveals that while AI technologies play a crucial role in detecting risks and predicting potential incidents, significant gaps remain in real-time intervention and recovery strategies. Most AI applications focus on probability estimation and detection rather than active prevention or automated response mechanisms. There is a need for more integrated, multi-sensor systems that can not only identify hazards but also take immediate corrective actions, such as stopping machinery, alerting operators, or adjusting equipment settings to prevent incidents. Additionally, recovery-focused AI solutions, such as those aiding in emergency response and post-incident analysis, are notably underdeveloped.

## 4. Discussion

The increasing application of AI across industries has been described as part of the Fourth Industrial Revolution, where automation, machine learning, and intelligent systems enhance efficiency, safety, and decision-making processes [38]. Innovations in artificial intelligence using sensors, robots, and ML algorithms have been shown to increase productivity and improve the safety and health of workers in the workplace. In agriculture, despite tremendous efforts to improve safety, the number of tractor-related incidents remains high [3].

In occupational safety and health (OSH), AI-driven solutions—such as sensor-based monitoring, predictive analytics, and automated interventions—have been shown to reduce workplace hazards by providing real-time risk detection and prevention strategies.

Traditional safety models, such as the Swiss cheese model, conceptualize incident prevention through multiple layers of defense, where failures in one layer can be mitigated by safeguards in others. However, these models are largely retrospective and do not dynamically adjust to real-time risks. Additionally, the Swiss cheese model lacks mechanisms for real-time monitoring of risk transitions and automated interventions. It does not integrate AI-driven predictive analytics to assess risk probability dynamically. Furthermore, it does not provide structured post-incident learning strategies to refine future risk mitigation approaches. These limitations reduce its effectiveness in rapidly evolving environments, such as agricultural operations, where conditions and hazards can change unpredictably. In contrast, the REDECA framework builds on this foundation by integrating AI-driven predictive capabilities to continuously monitor transitions between risk states (R1, R2, R3). By doing so, REDECA provides a proactive approach to workplace safety, ensuring that potential hazards are detected and mitigated before incidents occur.

In this paper, we extracted tractor-related fatality incidents from FACE reports on the CDC website and applied the REDECA framework to analyze pre-incident processes and identify potential AI solutions to mitigate these incidents. Our analysis led to several AI-driven recommendations aimed at reducing these fatalities and enhancing tractor operator safety. For run-over incidents, AI-based sensor technology can be implemented to alert drivers to turn off tractors before dismounting and to prevent the engine from starting while the operator is on the ground. Additionally, smart technology can ensure tractors remain in a parked state during maintenance, while pressure sensors could be integrated to detect and warn drivers against leaving their seats while the tractor is in operation. AI-driven systems could also prevent tractors from starting unless they are in neutral, ensure seat belt engagement before operation, and enhance safety mechanisms such as roll-over protective structures. Intelligent transmission systems should require tractors to be placed in park before operators dismount for equipment adjustments. For pinned by/crushed incidents, there is a significant gap in AI technology for detecting transitions between pre-incident and incident stages, minimizing damage and recovery time, and preventing entry into high-risk zones. AI solutions should be developed to detect hazardous conditions in real time and provide early warnings to prevent operators from entering dangerous scenarios. AI-enhanced driver assistance systems could help mitigate fatigue-related accidents by monitoring operator alertness and providing real-time recommendations for rest. Additionally, AI-powered emergency communication devices could enable rapid response in case of injury, reducing fatality risks. For entanglement incidents, AI-based safety wearables could detect entanglement hazards and enhance operator response in emergency situations. Intelligent control systems could ensure clutch, and brake pedals remain slip-resistant to improve control, while AI-powered automated inspections could identify potential hazards related to moving parts. Smart machine guarding systems could provide real-time protection by detecting proximity to hazardous components and automatically shutting down equipment when necessary.

For fall incidents, AI-driven monitoring could detect instability in tractor seats and alert users when attachments are loose. AI-based design optimization could improve step and handrail configurations for safer mounting and dismounting, while real-time health monitoring systems could assess operator wellness and issue alerts when medical intervention is necessary to prevent falls due to health conditions. AI-driven fatigue detection could also assess operator drowsiness and provide rest recommendations. For fire incidents, AI-integrated vehicle monitoring systems could continuously assess operational conditions, detect abnormalities that may lead to fires, and provide predictive alerts to operators. AI-powered thermal sensors could monitor temperature fluctuations in engine components and issue early warnings to prevent overheating. Additionally, AI-driven emergency response systems could activate automated fire suppression mechanisms and alert emergency services when fire risks are detected. For roll-over and overturn incidents, our analysis categorized cases into various classes, identifying Class 5—where tractors lose stability on irregular surfaces—as the most prevalent. AI-based terrain assessment technology could provide real-time stability analysis and adaptive warnings for operators driving on uneven ground. Additionally, AI-integrated load management systems could assist operators in maintaining proper weight distribution, reducing the likelihood of instability. Overturn incidents, often caused by adverse weather conditions or steep terrain, require AI-driven predictive systems to assess environmental conditions and alert operators to potential hazards before an incident occurs. AI-based training simulations could enhance operator awareness of roll-over risks by simulating hazardous scenarios and reinforcing safe operational practices. AI-assisted communication tools could ensure remote workers remain connected in case of emergencies. AI-driven counterweight management systems could dynamically adjust front-end weights to improve tractor stability, while intelligent fatigue monitoring could detect signs of exhaustion and prompt operators to take breaks. Across all incident types, common safety concerns highlight the necessity for AI-enhanced seat belt compliance detection, automated tractor inspections to identify missing or faulty safety features, and AI-powered medical monitoring for operators with pre-existing health conditions. AI-driven speed regulation systems could ensure tractors operate within safe speed limits, while AI-based hazard detection systems could continuously scan work environments to identify and warn operators about potential risks. Intelligent task assignment algorithms could match young workers with age-appropriate duties, and AI-enhanced road safety systems could provide adaptive grading recommendations or deploy warning signals at hazardous intersections. Furthermore, AI-controlled braking systems could prevent tractor instability in slippery conditions by dynamically adjusting braking mechanisms.

As noted throughout our analysis, there are few AI solutions for reducing recovery time, detecting transitions between R2 and R3, or intervening to minimize damage and facilitate recovery in R3 in different tractor related incident cases. This highlights a crucial area for future research and development. Enhancing AI-driven predictive models, integrating real-time monitoring systems, and leveraging advanced robotics for emergency response could help bridge this gap. Additionally, training farmers to adopt AI-based safety technologies, such as wearable tracking devices and emergency alert applications, is essential. Increasing awareness and trust in these technologies can improve response times and ensure timely assistance in critical situations, ultimately reducing the severity of injuries and fatalities.

While we have demonstrated the utility of REDECA in identifying hazards when operating tractors, we acknowledge several limitations of the study. For one, developing the probabilities for transitions among states R1, R2, and R3 can be time-intensive and difficult. In addition, the cost of developing AI solutions could be both cost-prohibitive and technologically infeasible. Moreover, it is unclear how well such solutions would integrate into current work processes and whether they would be perceived as disruptive despite potential benefits to safety and occupational health.

For example, one of the authors of this study was personally involved in a research study observing the initial sociotechnological integration of positive train control (PTC) immediately after the Rail Safety Improvement Act of 2008 was enacted [39]. Despite the benefits of using PTC to detect and prevent several severe railway hazards using AI, there was massive pushback against implementing it. For one, the cost to implement was approximately the same as the rail maintenance budget during the time of implementation. The technology also required many years to develop and verify, including establishing the infrastructure to operate the system. Rail culture also needed to change from very punitive to collegial before train operators trusted the system. Despite these, PTC was integrated into passenger rail and continues to operate as planned. The system also gathers data that can be used to establish hazards.

Given the potential benefits of using AI in tractor operations, we encourage future researchers to examine the hazards we have identified. Future research should also focus on integrating near miss incidents into the analysis. These incidents, which often reflect early warning signs of potential hazards, could enhance the predictive capabilities of the REDECA framework. By analyzing near miss data, AI models can be trained to detect patterns and implement proactive interventions before risks escalate to R3 (incident). Collaborating with agricultural stakeholders to systematically record and analyze near miss events could help establish a more comprehensive dataset for improving the effectiveness of AI-driven safety solutions. These include how to integrate technologies that can gather data about hazards in tractor operations cost-effectively, with minimal process disruptions. The potential savings preventing injury would be well worth the endeavors.

## 5. Limitations

While AI-driven solutions offer significant potential to enhance agricultural tractor safety, several feasibility and adaptability challenges must be considered:

1. High Costs: Implementing AI-based safety interventions often requires substantial financial investment in hardware (e.g., sensors, automated controls) and software (e.g., machine learning models, predictive analytics). Many small-scale farms may struggle to afford these technologies [39].
2. Technical Limitations: AI models rely on high-quality, real-time data to function effectively. Agricultural environments, with varying terrains and weather conditions, can introduce noise and unpredictability that limit AI accuracy. Additionally, maintaining AI-integrated machinery requires technical expertise, which may not be readily available in rural farming communities [40].
3. Adoption Resistance: Many farmers may be hesitant to adopt AI-based solutions due to unfamiliarity with the technology, concerns about reliability, and skepticism about its effectiveness. Integrating AI into traditional farming practices requires significant behavioral and cultural shifts [41].
4. Regulatory Challenges: The use of AI in agriculture, particularly for safety applications, may face regulatory scrutiny regarding data privacy, liability in case of AI-driven errors, and compliance with existing occupational safety laws. Regulatory frameworks must evolve to accommodate AI-based interventions while ensuring worker safety and accountability [42].
5. Data Limitations: Our study relied on FACE reports, which, while comprehensive, may not capture all tractor-related incidents. Variations in how reports are indexed and described could lead to potential false negatives, with relevant cases being unintentionally excluded. Future studies could mitigate this by employing natural language processing (NLP) techniques to improve data extraction and analysis [43].

## 6. Conclusions

AI technology has been successfully spread across all industries, including agriculture, mining, transportation, construction, and more. The use of AI technology has shown significant improvement in the health and safety of workers in the workplace. REDECA is a useful framework that highlights AI/OSH strengths and opportunities for advances in sensors, robotics, and machine learning algorithms to improve working conditions in the workplace. Given the high and steady fatal incident rate within agricultural workplaces related to tractor drivers, we used the REDECA framework to deeply understand every step involved before and after the occurrence of the incidents among tractor drivers and to identify existing AI solutions used to improve the safety of the tractor drivers. Moreover, the REDECA framework helped identify specific areas where AI solutions are missed and can be developed to reduce the occurrence of incidents and recovery time. Based on our findings, key AI-driven interventions include real-time sensor-based monitoring for hazard detection, predictive analytics for early risk identification, and automated intervention systems to prevent accidents. Additionally, AI-powered emergency response mechanisms can reduce recovery time by rapidly alerting medical services. These targeted AI solutions can bridge existing gaps in agricultural safety, ultimately improving tractor driver health and reducing fatal incidents.

## Supporting information

S1 Table REDECA framework results summary for run over cases. (DOCX)

S2 Table REDECA framework results summary for Pinned by cases. (DOCX)

S3 Table REDECA framework results summary for fall cases. (DOCX)

S4 Table REDECA framework results summary for others including fires and crashes. (DOCX)

S5 Table REDECA framework results summary for roll over cases. (DOCX)

S6 Table REDECA framework results summary for overturn cases. (DOCX)
