# Supplementary material for: AI-driven solutions to improve safety and health: Application of the REDECA framework for agricultural tractor drivers
Source: PLOS Glob Public Health. 2025 Jun 4;5(6):e0003543. doi: 10.1371/journal.pgph.0003543 (PMC12136288; doi:10.1371/journal.pgph.0003543)
Supplement: S1 Table — (DOCX) [file pgph.0003543.s001.docx]

|  | **R1** | **R2** | **R3** |
| --- | --- | --- | --- |
| Description | On the ground | The driver is at risk of run over while driving. | 1. Run over due to unsecured seat placement. 2. Run over due to the tractor left in gear after its last use. 3. Run over because of the weight of the water tank trailer and high speed. 4. Run over because the tractor engine was operating at high rpm, slipped into gear, and accelerated forward at high speed. 5. Run over due to the wheels on the right side of the tractor running over a tree stump. 6. Run over due to the tractor being in forward gear and the driver standing on the ground trying to turn the tractor on. 7. Run over due to tractor being left on a hill while running without driver and dismounted from its initial attached loader bucket. 8. Run over due to driver having a seizure and falling out off the tractor because they did not use a seat belt. 9. Run over due to unintended door opening causing the driver to fall out of the tractor. 10. Run over due to non-functional brake. 11. Run over due to non-functional gear. 12. Run over due to the driver falling off from the tractor moving at high speed. 13. Run over due to a driver falling off the tractor while moving. The driver tried to jump on the tractor, slipped and engaged the gear making the tractor move while the victim was on the ground. 14. Run over due to falling off the tractor while making a sharp turn. 15. Run over due to poor driver visibility. |
| **AI-based Solutions** | | | |
| Probability of entering next stage | NOT APPLICABLE: 100% the driver will seat on the tractor seat. | (1) Vibration sensor [19]  (2) Sensor alerts turn off tractor after removing the seatbelt [20]  (3) Sensor alerts overweight and high speed. Augmented Reality (AR) shows speed on windshield [21].  (4) Sensor alerts turn off tractor after removing seatbelt. Sensor alerts for overweight and high speed. AR shows speed on windshield [21].  (5) AR identifies obstacles in front. Sensor detects and avoid obstacles [22].  (6) Pressure sensor prevents tractor from starting unless enough pressure is on the seat [23]  (7) Sensor alerts turn off tractor after removing the seatbelt [20].  (8) Sensor alerts user and prevents tractor from moving without seatbelt being on [20].  (9) Sensor to lock the door of the tractor door while moving [24]  (10) Sensor alerts user when brake is non-functional [21]  (11) Sensor alerts user when gear is non-functional [21]  (12) Sensor alerts user about ROPS, seatbelt, and high speed. Sensor alerts user when falls happen [25].  (13) Fall sensors [26].  (14) Fall sensors [27].  (15) AR and wearable sensors alert people when potential hazards happen [28]. | NOT APPLICABLE: There is not any stage after this stage (R3). |
| Probability of reduced recovery time | NOT APPLICABLE: Actual hazard has not occurred. | NOT APPLICABLE: Actual hazard has not occurred. | None |
| Detect change between stages | NOT APPLICABLE: From R1 to R2, driver leaves ground to sit in tractor seat. | (1) Vibration sensor  (6) Pressure sensor prevent tractor from starting unless enough pressure is on the seat [23]. | NOT APPLICABLE: No stage after R3. |
| Intervention prevents entry to next stage | NOT APPLICABLE: The driver should mount and sit on the tractor. | (2) Sensor alerts user to turn off tractor after removing seatbelt [21].  (3) Sensor alerts when overweight and high speed. AR shows speed on windshield [21].  (4) Sensor alerts when overweight and high speed. AR shows speed on windshield. Sensor alerts user to turn off tractor after removing seatbelt [21]  (5) AR on mirrors show distance from tractor to obstacle. Warns driver of hazard. Sensor detects and avoids obstacles [29].  (6) Pressure sensors to prevent tractor from starting unless enough pressure is on the seat [23].  (7) Sensor alerts user to turn off tractor after removing the seatbelt [21].  (8) Sensor alerts user and prevents tractor movement without seatbelt being on [21].  (9) Sensor prevents opening tractor door while moving [23].  (10) Sensor alerts user when brake is non-functional [21].  (11) Sensor alerts user when gear is non-functional [21].  (12) Sensor alert user about ROPS, seatbelt, and high speed [21]. | NOT APPLICABLE: There is not any other stage after this. |
| Intervention sends worker to previous stage | NOT APPLICABLE: No stage before R1. | NOT APPLICABLE: Driver needs to be seated on tractor seat. | None |
| Intervention to minimize damage and recovery | NOT APPLICABLE: Hazard has not occurred. | NOT APPLICABLE: The hazard has not occurred. | (12) Wearable sensor like a smart watch signals rescue in case of fall. [30]  (13) Wearable sensor like a smart watch signals rescue in case of fall. [31]  (14) Wearable sensor like a smart watch signals rescue in case of fall. [32]  (15) Wearable sensor like a smart watch signals rescue in case of fall. [33] |
