# Supplementary material for: AI-driven solutions to improve safety and health: Application of the REDECA framework for agricultural tractor drivers
Source: PLOS Glob Public Health. 2025 Jun 4;5(6):e0003543. doi: 10.1371/journal.pgph.0003543 (PMC12136288; doi:10.1371/journal.pgph.0003543)
Supplement: S2 Table — (DOCX) [file pgph.0003543.s002.docx]

|  | **R1** | **R2** | **R3** |
| --- | --- | --- | --- |
| Description | On the ground | Driver at risk of being pinned while driving. | 1. Pinned between the enclosed auger and the tractor steering wheel due to an accident between the tractor and barn wall. 2. Pinned to tree while inside tractor cab. 3. Pinned by a truck bed attached to a tree by a chain. 4. Pinned by and compressed by an attachment while trying to detach it. 5. Pinned by a tractor that drove into a ditch where the victim was located. 6. Pinned by hayrack roll over that pinned the victim to the left rear tractor tire. |
| **AI-based Solutions** | | | |
| Probability of entering next stage | NOT APPLICABLE: 100% driver sits on tractor seat. | (1) AR and perception sensors [51]  (2) AR and perception sensors [52]  (3) AR and perception sensors [55]  (4) AR and perception sensors [62]  (5) AR and perception sensors [51]  (6) AR and perception sensors [52] | NOT APPLICABLE: No stage after R3. |
| Probability of reduced recovery time | NOT APPLICABLE: Hazard has not occurred. | NOT APPLICABLE: Hazard has not occurred. | None |
| Detect change between stages | NOT APPLICABLE: From R1 to R2, the driver leaves ground to sit on tractor seat. | None | NOT APPLICABLE: No stage after R3. |
| Intervention to prevent entry to next stage | NOT APPLICABLE: Driver mounts tractor and sits in it. | None | NOT APPLICABLE: No stage after this. |
| Intervention to send worker to previous stage | NOT APPLICABLE: No stage before R1. | NOT APPLICABLE: Driver needs to sit in tractor seat. | None |
| Intervention to minimize damage and recovery | NOT APPLICABLE: Hazard has not occurred. | NOT APPLICABLE: Hazard has not occurred. | None |
