# Supplementary material for: AI-driven solutions to improve safety and health: Application of the REDECA framework for agricultural tractor drivers
Source: PLOS Glob Public Health. 2025 Jun 4;5(6):e0003543. doi: 10.1371/journal.pgph.0003543 (PMC12136288; doi:10.1371/journal.pgph.0003543)
Supplement: S3 Table — (DOCX) [file pgph.0003543.s003.docx]

|  | **R1** | **R2** | **R3** |
| --- | --- | --- | --- |
| Description | On the ground | Driver at risk for fall while driving. | 1. Fall out of the tractor while driving due to an unattached seat. 2. Fall off due to slip on a tractor step. 3. Fall off due to tractor driving at high speed on a incline and driver not wearing a seatbelt. 4. Fall off from the tractor due to heart issues and not wearing a seatbelt. 5. Fall off due to tractor jumping jack-knifed causing the driver to be thrown off from the tractor. 6. Fall from tractor due to loss of control while driving. |
| **AI-based Solutions** | | | |
| Probability of entering next stage | NOT APPLICABLE: 100% driver sits on the tractor seat.  No AI solution to prevent driver from sitting in the tractor. | 1.Vibration sensors [19]  2.Fall sensors [24]  3. Augmented reality [26]  4. Fall sensors [27]  5. Fall sensors [28]  6. Fall sensors [32] | NOT APPLICABLE: No stage after R3. |
| Probability of reduced recovery time | NOT APPLICABLE: Hazard has not occurred. | NOT APPLICABLE: Hazard has not occurred. | None |
| Detect change between stages | NOT APPLICABLE: From R1 to R2, driver leaves ground to sit on tractor seat. | None | NOT APPLICABLE: No stage after R3. |
| Intervention to prevent entry to next stage | NOT APPLICABLE: Driver should mount tractor and seat in it. | None | NOT APPLICABLE: No other stage after this. |
| Intervention to send worker to previous stage | NOT APPLICABLE: No stage before R1. | NOT APPLICABLE: Driver needs to sit in tractor seat. | None |
| Intervention to minimize damage and recovery | NOT APPLICABLE: Hazard has not occurred. | NOT APPLICABLE: Hazard has not occurred. | 1.2.3.4.5.6. Fall sensors |
