# Supplementary material for: AI-driven solutions to improve safety and health: Application of the REDECA framework for agricultural tractor drivers
Source: PLOS Glob Public Health. 2025 Jun 4;5(6):e0003543. doi: 10.1371/journal.pgph.0003543 (PMC12136288; doi:10.1371/journal.pgph.0003543)
Supplement: S4 Table — (DOCX) [file pgph.0003543.s004.docx]

|  | **R1** | **R2** | **R3** |
| --- | --- | --- | --- |
| Description | On the ground | Driver at risk of different hazards while driving. | 1. Victim struck by hay bale that fell out of elevated bucket. 2. Victim fell while re-attaching a shaft to a tractor due to control lost in the hollow. 3. Entrapment due to victim’s shirt entrapped inside an auger not equipped with guard. 4. Entanglement due tractor left on and hay baler left on downward slope. 5. Incident due to a tractor driver hauling a tree higher than the recommended height. 6. Crash due to a semi-truck hitting a tractor causing both victims to be ejected from their respective vehicles. 7. Crash due to a detached wagon moving forward, crushing the user against the tractor. 8. Fire due to the victim puncturing an above ground gas line with a tractor due to poor visibility. 9. Fire due to vinyl shrouds bursting into flames, spreading to the victim’s clothing. 10. Fire due to ignition of a tractor struck by a tree. |
| AI-based Solutions | | | |
| Probability of entering next stage | NOT APPLICABLE: 100% driver sits in tractor seat.  No AI solution to prevent driver sitting in tractor. | 1. Fall sensors [28]  2. Fall sensors [26]  3. [34]  4. Sensor prevents user from leaving tractor on.  5. Height sensors [35]  6. Augmented reality  7. [22]  8. [34]  9. [22]  10.Augmented reality | NOT APPLICABLE: No stage after R3. |
| Probability of reduced recovery time | NOT APPLICABLE: Hazard has not occurred. | NOT APPLICABLE: Hazard has not occurred. | None |
| Detect change between stages | NOT APPLICABLE: From R1 to R2, driver leaves ground to sit in tractor seat. | None | NOT APPLICABLE: No stage after R3. |
| Intervention to prevent entry to next stage | NOT APPLICABLE: Driver should mount tractor and sit in it. | None | NOT APPLICABLE: No stage after R3. |
| Intervention to send worker to previous stage | NOT APPLICABLE: No stage before R1. | NOT APPLICABLE: Driver needs to sit in tractor seat. | None |
| Intervention to minimize damage and recovery | NOT APPLICABLE: Hazard has not occurred. | NOT APPLICABLE: Hazard has not occurred. | 1. Fall sensors [32]  2. Fall sensors [30] |
