# Supplementary material for: AI-driven solutions to improve safety and health: Application of the REDECA framework for agricultural tractor drivers
Source: PLOS Glob Public Health. 2025 Jun 4;5(6):e0003543. doi: 10.1371/journal.pgph.0003543 (PMC12136288; doi:10.1371/journal.pgph.0003543)
Supplement: S5 Table — (DOCX) [file pgph.0003543.s005.docx]

|  | **R1** | **R2** | **R3** |
| --- | --- | --- | --- |
| Description | On the ground | Driver at risk of different hazards while driving. | 1. Roll over due to a tractor’s sharp turn into a ditch. 2. Roll over due to tractor driving and reaching a steep roadside. 3. Roll over due de-attaching a wagon from the tractor on a hilly, sharply curved road. 4. Roll over due to an attached overweight hay bale and driving on a slope. 5. Roll over due to tractor driving on an irregular surface causing its center of gravity to shift resulting in tractor instability. 6. Roll over due to a rasied tractor bucket, and attempting to make a left turn while on the incline. 7. Roll over due to driver foot slipping off the tractor’s clutch while trying to pull out another tractor stuck in mud. 8. Roll over due to the tractor being stopped and dismounted without setting the brakes and leaving the manual transmission in gear. 9. Roll over due to the tractor not equipped with ROPS and seat belt reaching an embankment while the brakes were not engaged. |
| **AI-based Solutions** | | | |
| Probability of entering next stage | NOT APPLICABLE: 100% driver sits in tractor seat.  No AI solution to prevent driver sitting in tractor. | 1.Perception sensors [24]  2.Augmented reality  3. [20]  4.Weight Sensor  5. [24]  6. [22]  7. [34]  8. Sensor prevents leaving transmission in gear.  9. Identify non-functional brake | NOT APPLICABLE: No stage after R3. |
| Probability of reduced recovery time | NOT APPLICABLE: 100% driver sits in tractor seat. | NOT APPLICABLE: Hazard has not occurred. | None |
| Detect change between stages | NOT APPLICABLE: Hazard has not occurred. | None | NOT APPLICABLE: No stage after R3. |
| Intervention to prevent entry to next stage | NOT APPLICABLE: From R1 to R2, the driver leaves ground to sit in tractor seat. | None | NOT APPLICABLE: No stage after R3. |
| Intervention to send worker to previous stage | NOT APPLICABLE: Driver should mount tractor and sit in it. | NOT APPLICABLE: Driver needs to sit in tractor seat. | None |
| Intervention to minimize damage and recovery | NOT APPLICABLE: No stage before R1. | NOT APPLICABLE: Hazard has not occurred. | None |
