# Supplementary material for: AI-driven solutions to improve safety and health: Application of the REDECA framework for agricultural tractor drivers
Source: PLOS Glob Public Health. 2025 Jun 4;5(6):e0003543. doi: 10.1371/journal.pgph.0003543 (PMC12136288; doi:10.1371/journal.pgph.0003543)
Supplement: S6 Table — (DOCX) [file pgph.0003543.s006.docx]

|  | **R1** | **R2** | **R3** |
| --- | --- | --- | --- |
| Description | On the ground | Driver at risk of different hazards while driving. | 1. Overturn due to wet/snow/muddy hill causing the tractor to slide down. 2. Overturn due to tractor driving on slope terrain and ditch. 3. Overturn due to tractor driving on the right side of the road with a heavy trailer was connected and attempting to overcorrect. 4. Overturn due to the tractor turning on a slope/hill. 5. Overturn due to the tractor sliding down a steep embankment. 6. Overturn due to the tractor attached to overweight objects. 7. Overturn due to the tractor wheel coming off the ground while driving. 8. Overturn due non-functional gear shift. 9. Overturn due tractor trying to pull out another tractor stuck into mud. 10. Overturn due to speeding tractor |
| **AI-based Solutions** | | | |
| Probability of entering next stage | NOT APPLICABLE: 100% driver sits in tractor seat. | 1. [22]  2. Perception sensor [28]  3. Weight sensor [20]  4. [34]  5.  6.Weight sensor [20]  7.Losing wheel sensor  9.  8.Non-functional gear sensor  9.  10.Speeding sensor [21] | NOT APPLICABLE: There is not any stage after this stage (R3). |
| Probability of reduced recovery time | NOT APPLICABLE: 100% driver sits in tractor seat. | NOT APPLICABLE: Hazard has not occurred. | None |
| Detect change between stages | NOT APPLICABLE: Hazard has not occurred. | None | NOT APPLICABLE: No stage after R3. |
| Intervention to prevent entry to next stage | NOT APPLICABLE: From R1 to R2, the driver leaves ground to sit in tractor seat. | None | NOT APPLICABLE: Not stage after R3. |
| Intervention to send worker to previous stage | NOT APPLICABLE: Driver mounts tractor and sits in it. | NOT APPLICABLE: Driver needs to sit in tractor seat. | None |
| Intervention to minimize damage and recovery | NOT APPLICABLE: No stage before R1. | NOT APPLICABLE: Hazard has not occurred. | None |
